# Improving Acute Treatment of Alcohol Withdrawal at an Inpatient De-addiction Ward (‘Vimukthi’) at Kerala State, India: Full Cycle of a Clinical Audit

**DOI:** 10.1192/bjo.2024.100

**Published:** 2024-08-01

**Authors:** Achu S, Ajay Kuriakose, Ramkumar Sathiaseelan

**Affiliations:** 1 Government Medical College Idukki, Idukki, India; 2 Vimukthi Deaddiction Centre, Idukki, India

## Abstract

**Aims:**

Long-acting benzodiazepine is the treatment standard for alcohol withdrawal and three regimens are defined – fixed-dose (for outpatient and inpatient settings with untrained staff), symptom-triggered (inpatient setting with trained staff) and front-loading (when a severe withdrawal state is anticipated). Standards in this regard are published by ASAM, NICE and guidelines by Govt. of India. A clinical audit was performed to explore the treatment strategy used in a de-addiction centre in India.

**Methods:**

Description of the initial audit cycle.

*Setting:* Dedicated 10-bed de-addiction ward, attached to a general hospital, with an average of 15 admissions/month of patients with disorders of alcohol use. The centre was established as a special project (‘Vimukthi’) in 2018 and is serviced by a team of three nurses, one doctor and one clinical psychologist, and visited by psychiatrists from the general hospital.

*Measurement of performance and comparison with standards*: Measurement was done in May 2023 after the authors took charge of the ward. The centre used a fixed-dose regimen of short-acting lorazepam for all patients to manage withdrawal symptoms. There was no documentation of risk profiling. We therefore recommended that tailored treatment based on patient profile be introduced. Risk profiling based on symptoms, signs and history and a symptom-triggered regimen for withdrawal management using nurse-administered CIWA-AR rating could be incorporated into a standard operating procedure (SOP). An SOP was developed and after team discussion and training it was introduced in October 2023.

**Results:**

Re-audit of the implementation phase of SOP over three months (Oct 2023 to Dec 2023) was conducted. Case files were noted to document risk stratification as 34% low risk, 52% intermediate risk and 14% high risk. Symptom-triggered regimen was administered to all patients with added front-loading for all high-risk and some moderate-risk patients. Staff and patients expressed satisfaction with the new protocol. We noticed a significant reduction in the use of oral lorazepam (from 3324 mg for 63 patients during the comparative period of Oct 2022–Dec 2022 to 10 mg for 39 patients), while the use of injectable lorazepam increased by 25% (0.8 mg/patient to 1 mg/patient). Use of oral diazepam increased from nil to 170 mg with one patient receiving injectable diazepam.

**Conclusion:**

Introducing an SOP that incorporated risk profiling, use of long-acting benzodiazepines, symptom-triggered and front-loading regimens and nurse-administered CIWA-Ar monitoring led to the reduced use of short-acting and uptake of long-acting oral benzodiazepines in inpatient alcohol withdrawal management. Decisions based on risk profiling led to an increase in the use of injectable benzodiazepines. We report that conducting this audit cycle led to the improvement of treatment standards in a specialized inpatient de-addiction centre in India.

